# Diagnostic value of platelet to high-density lipoprotein cholesterol ratio in abdominal aortic aneurysms

**DOI:** 10.3389/fcvm.2025.1687265

**Published:** 2025-10-30

**Authors:** Xiao Du, Jin Xu, Shilan Zhang, Ling Liu

**Affiliations:** ^1^ Department of Cardiology, Beijing Friendship Hospital, Capital Medical University, Beijing, China; ^2^ Department of Cardiovascular Medicine, The Second Xiangya Hospital, Central South University, Hunan, China; ^3^ Department of Cardiology, Songjiang Hospital and Songjiang Research Institute School of Medicine, Shanghai Jiao Tong University, Shanghai, China

**Keywords:** abdominal aortic aneurysm, PHR, diagnostic marker, thrombosis, platelet, high-density lipoprotein cholesterol

## Abstract

**Background:**

Abdominal aortic aneurysm (AAA) is a disease with a relatively high mortality risk. Currently, diagnosis of AAA predominantly depends on imaging examinations, and there is an urgent need for simple and rapid screening biomarkers. The platelet to high-density lipoprotein cholesterol ratio (PHR) is an emerging biomarker reflects systemic inflammation and hypercoagulable states in cardiovascular diseases. The aim of this study is to elucidate the clinical significance of PHR in AAA.

**Methods:**

A total of 156 patients with AAA and 113 healthy controls were enrolled. Among them, 80 patients with AAA had concomitant thrombosis. Serum samples were collected upon admission to the hospital and before treatment. The equation for PHR calculation is: PHR = [platelet (1,000 cells/µl)/HDL-C (mmol/L)]. The optimal PHR cut-off was determined via ROC analysis, and logistic regression identified AAA predictors.

**Results:**

In this study, PHR level in patients with AAA was significantly higher than that of control group [127.00 (109.74, 163.42) vs. 192.35 (159.08, 231.66); <0.001]. Additionally, PHR value was even higher in AAA patients with thrombosis [161.80 (135.54, 193.41) vs. 220.61 (186.49, 262.27); <0.001]. ROC curve analysis revealed that PHR had a good predictive value for diagnosis of AAA, with an area under the curve (AUC) of 0.805. When it came to the diagnosis of AAA patients with thrombosis, the AUC of PHR was 0.814. Multivariate logistic regression analysis further demonstrated that PHR was an independent predictor in AAA (OR: 1.022, 95% CI: 1.012–1.031; *P* < 0.001) and AAA patients with thrombosis (OR: 1.016; 95% CI: 1.007–1.024; *P* < 0.001).

**Conclusion:**

PHR has good diagnostic value for AAA and can serve as a rapid screening indicator for its clinical diagnosis.

## 1 Background

Abdominal aortic aneurysm (AAA) refers to a localized, progressive dilation of the abdominal aorta with a diameter ≥3 cm or exceeding 50% of the normal value (1). AAA typically manifests as a clinically occult condition, and patients often remain asymptomatic until the disease progresses (2). However, its rupture can lead to a mortality rate of more than 80%, representing a life-threatening aortic emergency (3, 4). Due to the high risk of rupture and associated mortality, timely diagnosis and personalized management of AAA are essential. Currently, the diagnosis of AAA mainly relies on imaging examination methods such as ultrasound, computed tomography angiography (CTA), or magnetic resonance angiography (MRA) (5, 6). In regions with limited resources or in urgent clinical situations, these examination methods may encounter difficulties such as high costs and poor accessibility. Over recent years, several serum biomarkers for AAA progression have been investigated, including D-dimer, matrix metalloproteinases (MMPs), C-reactive protein (CRP), inflammatory cytokines (e.g., IL-6, TNF-α) (1, 7–9). However, these biomarkers often exhibit limitations. For instance, they have relatively low sensitivity in diagnosing AAA, or are difficult to be widely applied in clinical practice. In recent years, there has been a notable increase in the number of biomarkers identified for evaluating the growth of AAAs. Among these, research has highlighted that elevated levels of circulating trimethylamine N-oxide (TMAO) are associated with a heightened risk of AAA development and can serve as a predictor for rapid AAA expansion (10). Despite these findings, TMAO has not yet gained widespread adoption in clinical practice. Therefore, it is an urgent priority to explore new biomarkers for the early screening of AAA, which can contribute to early intervention in AAA and thus prevent the occurrence of adverse events.

Abnormal lipid metabolism plays an important role in the occurrence and development of AAA (1, 11). High-density lipoprotein cholesterol (HDL-C), often termed the “good cholesterol”, inhibits the occurrence and development of AAA by facilitating reverse cholesterol transport to remove lipids from the arterial walls, inhibiting oxidative stress responses, and regulating immune responses (1, 11, 12). Clinical studies have shown that low HDL-C levels are independently associated with an increased risk of AAA (13). Moreover, platelets can also play a crucial role in the pathogenesis of AAA through multiple mechanisms, including participation in hemostasis and thrombosis, triggering of inflammatory responses, promotion of vascular remodeling, and degradation of the extracellular matrix, among others (14–16). Studies have demonstrated that treatment with low-dose aspirin is beneficial for patients with AAA, as it can reduce the volume of the aneurysms and decrease the risk of rupture of AAA (17, 18). A recent study demonstrated that the level of soluble glycoprotein VI (sGPVI) can predict the diagnosis of AAA and AAA growth rate, and may serve as a therapeutic target for AAA (19). Although sGPVI is not yet widely applied in clinical settings, its crucial role in platelet activation suggests that platelet-related indicators may have significant potential in predicting AAA.

The platelet to high-density lipoprotein cholesterol ratio (PHR), has garnered attention as a promising biomarker for AAA due to its integrative nature and practical clinical utility. The pathogenesis of AAA is multifaceted, encompassing both inflammatory and metabolic processes (20). Platelet activation and dyslipidemia are well-documented contributors to AAA development. Platelets play a crucial role in promoting inflammation and thrombosis, which are central to aneurysm formation and progression (21). In contrast, HDL-C exerts anti-inflammatory and anti-atherosclerotic effects. By amalgamating these two critical aspects, the PHR provides a composite measure that holistically reflects the interplay between inflammation and metabolism, both integral to AAA pathogenesis (22, 23).

Accumulating evidence underscores the association of PHR with various cardiovascular diseases, such as hypertension, heart failure, and stroke (20–23), and a retrospective multicenter study indicates that elevated PHR increases the long-term mortality risk in patients with CHD, serving as a high-risk marker to guide clinical intervention (24).Given the overlapping pathophysiological pathways between these conditions and AAA, it is plausible to hypothesize that PHR could serve as a valuable indicator for AAA. This hypothesis is further supported by the mechanistic linkages between platelet activation, lipid metabolism, and aneurysm development. Clinically, PHR stands out for its practicality and cost-effectiveness. It can be readily calculated from routine blood tests, making it easily accessible for widespread application across diverse healthcare settings (24). This accessibility is crucial for the routine monitoring and early detection of AAA, potentially enhancing clinical decision-making and patient outcomes.

PHR not only integrates key pathophysiological components of AAA but also offers a practical and readily available marker for clinical use, making it a compelling candidate for further investigation in the diagnosis and management of AAA. Therefore, this study aims to investigate the clinical application value of PHR in AAA, with the expectation of providing a rapid and simple laboratory-based method to assist in the diagnosis of AAA.

## 2 Methods

### 2.1 Study population

In this study, patients with AAA who were treated in the Emergency Department and the Department of Vascular Surgery of our hospital from January 2018 to December 2018 were consecutively enrolled. Among them, patients with Marfan syndrome, acute myocardial infarction or stroke, malignant tumors, severe liver and kidney insufficiency, active inflammatory diseases, or other connective tissue diseases were excluded from the study. A total of 169 patients with AAA were included in this study. Meanwhile, we recruited 120 gender-matched healthy individuals who underwent comprehensive health examinations in the same hospital as the control group. AAA was diagnosed by chest radiograph, transesophageal echocardiography, computed tomography, or surgery, and it was determined whether there was concomitant thrombosis. AAA was defined as segmental abnormal dilation of the abdominal aorta, with a diameter increase of more than 50% compared to the diameter of the normal aorta. All healthy control individuals in this study underwent imaging examinations to confirm the absence of AAA.

Among the initial 289 patients, 20 were excluded due to the lack of complete data on the variables required for this study. Finally, 156 patients with AAA and 113 healthy control individuals were included in our analysis. This study was approved by the Ethics Committee, and signed informed consent was obtained from all patients before enrollment. The study protocol adhered to the Declaration of Helsinki, and all participants provided written confirmation of their willingness to participate.

### 2.2 Data collection and definitions

We collected a variety of data from the patients' medical records, covering cardiovascular risk factors, demographic characteristics, past medical history, laboratory test indicators and other data. Diabetes can be diagnosed if any of the following criteria is met: random blood glucose ≥11.1 mmol/L; fasting blood glucose ≥7.0 mmol/L; blood glucose 2 h after the oral glucose tolerance test (OGTT) ≥ 11.1 mmol/L; or HbA1c ≥ 6.5%. The criteria for diagnosing hypertension are as follows: under resting conditions, systolic blood pressure (SBP) ≥ 140 mmHg or diastolic blood pressure (DBP) ≥ 90 mmHg, or the patient is currently receiving antihypertensive drug treatment. Coronary heart disease (CHD) is diagnosed according to relevant guidelines. In addition, the body mass index (BMI) is calculated by dividing the weight (in kg) by the square of the height (in m^2^).

### 2.3 Laboratory measurements

After the patients were admitted to the hospital, blood samples were collected from the anterior cubital veins before any medication was administered for the detection of multiple indicators. These indicators include blood routine tests, total cholesterol (TC); triglyceride (TG); low-density lipoprotein cholesterol (LDL-C); HDL-C, as well as serum creatinine, uric acid, blood urea nitrogen (BUN), etc. The calculation formula for the PHR is: PHR = [platelet (1,000 cells/µl)/HDL-C (mmol/L)]. All data were collected by professionals who were not involved in this study.

### 2.4 Statistical analysis

This study conducted statistical analyses using the Statistical Package for Social Sciences (SPSS) v.22. and GraphPad software 8.0.1. Continuous variables that conform to a normal distribution are represented by the mean ± standard deviation, and the *t*-test is applied for intergroup comparisons. Continuous variables that do not conform to a normal distribution are expressed as the median (interquartile range), and the Mann–Whitney *U*-test is chosen for intergroup comparisons. Categorical variables were presented in the form of counts and percentages (%), and the analysis of intergroup differences was completed through the Pearson's chi-squared (*χ*²) test or Fisher's exact test. The correlation between variables in patients with AAA was performed by Pearson or Spearman correlation analysis. The predictive value of the PHR for AAA and AAA with thrombosis was evaluated through the receiver operating characteristic (ROC) curve, and the optimal cut-off value of PHR was determined. Meanwhile, the sensitivity and specificity corresponding to each cut-off value were analyzed. Logistic regression analysis was used to screen the predictive factors for AAA and the predictive indicators for the presence of thrombosis in AAA patients. In the multivariate logistic regression model, variables with a *P* < 0.10 were included in the model. All statistical analysis was conducted two-sided, and the *P*-value < 0.05 was regarded as statistically significant.

## 3 Results

### 3.1 Baseline characteristics

A total of 269 patients were included in this study, including 156 patients with AAA and 113 gender-matched healthy controls. The baseline characteristics of the study population are presented in Table 1.

**Table 1:** Clinical characteristics of the study population.

Variables	Control	AAA	*P* value
General characteristics			
Age (years)	58.17 ± 9.12	60.39 ± 9.05	0.049
Male, *n* (%)	91 (80.5)	135 (86.5)	0.184
BMI (kg/m^2^)	23.52 ± 2.39	23.69 ± 2.52	0.593
Heart rate (beats per minute)	75.39 ± 10.69	80.04 ± 13.36	0.003
Comorbidities			
CHD, *n* (%)	11 (9.7)	24 (15.4)	0.174
Smoking, *n* (%)	34 (30.1)	59 (37.8)	0.188
Hypertension, *n* (%)	19 (16.8)	96 (61.5)	<0.001
Diabetes, *n* (%)	14 (12.4)	18 (11.5)	0.832
Laboratory values			
PHR	127.00 (109.74, 163.42)	192.35 (159.08, 231.66)	<0.001
Platelet count (×10^9^/L)	190.00 (154.50, 222.00)	186.00 (155.25, 225.00)	0.418
HDL-C (mmol/L)	1.41 (1.22, 1.62)	0.99 (0.84, 1.15)	<0.001
TG (mmol/L)	1.09 (0.82, 1.53)	1.31 (0.95, 2.03)	0.004
TC (mmol/L)	4.42 ± 0.71	4.47 ± 1.27	0.667
LDL-C (mmol/L)	2.45 ± 0.53	2.75 ± 1.07	0.002
WBC count (×10^9^/L)	5.63 (4.69, 6.79)	6.55 (5.33, 7.81)	<0.001
Serum creatinine (μmol/L)	74.30 (65.65, 81.50)	81.30 (67.78, 98.80)	<0.001
Uric acid (μmol/L)	325.30 (295.15, 368.40)	343.50 (298.15, 424.75)	0.464
BUN (mmol/L)	5.39 (4.56, 6.32)	6.34 (4.99, 7.48)	<0.001

AAA, abdominal aortic aneurysm; BMI, body mass index; CHD, coronary heart disease; PHR, platelet to high-density lipoprotein cholesterol ratio; HDL-C, high density lipoprotein cholesterol; TG, triglyceride; TC, total cholesterol; LDL-C, low density lipoprotein cholesterol; WBC, white blood cell; BUN, blood urea nitrogen.

**Figure 1:**
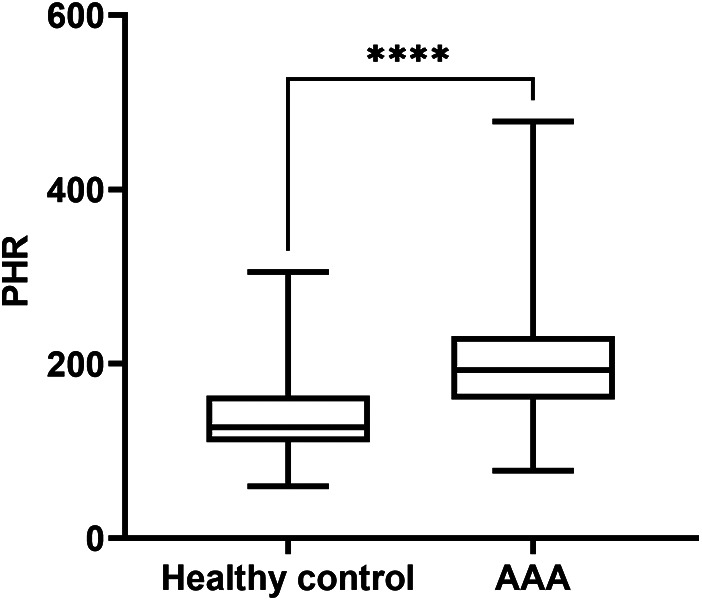
PHR levels in patients with AAA and healthy control group. Data were presented as median (min -max). AAA, abdominal aortic aneurysm; PHR, platelet to high-density lipoprotein cholesterol ratio; ****P < 0.0001.

Patients with AAA were significantly older than healthy controls, with a faster heart rate and a higher prevalence of hypertension. However, there were no significant differences in gender, BMI, or smoking history between the two groups, and the prevalences of diabetes and CHD were similar. In terms of laboratory values, AAA patients also had higher levels of TG, LDL-C, WBC, serum creatinine, and BUN. However, their HDL-C level was significantly lower, and there were no significant differences in platelet count, TC, and uric acid levels. Interesting, the PHR level in AAA patients was significantly higher [192.35 (159.08, 231.66) vs. 127.00 (109.74, 163.42); *P* < 0.001] (Figure 1). To further assess the significance of PHR levels in AAA patients across different platelet count strata, we stratified all participants into two groups: one with platelet counts exceeding the group's mean (designated as the high platelet count group) and another with counts below this mean (low platelet count group). This stratification was based on the average platelet count determined for each participant group. Our analysis revealed a notable elevation in PHR across both AAA patient groups, as detailed in Additional File 1: Supplementary Figure S1.

### 3.2 The correlation between PHR and aneurysm diameter in patients with AAA

**Figure 2:**
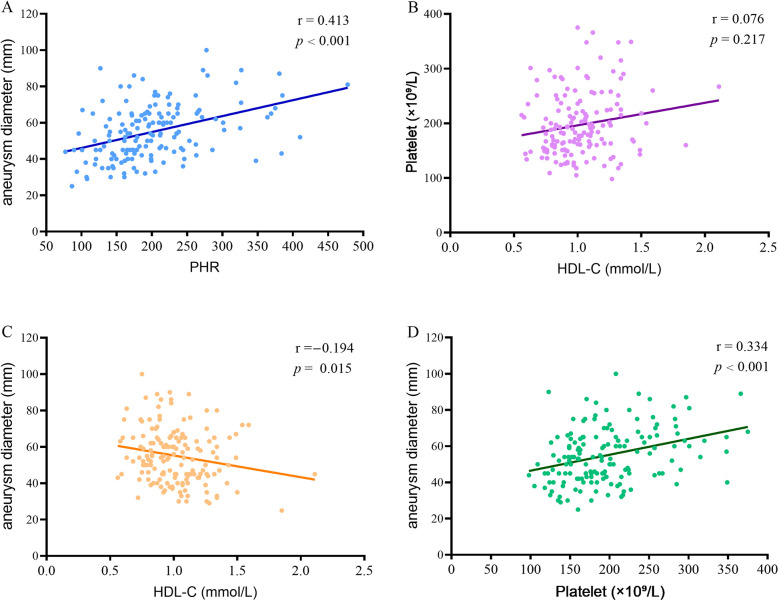
Correlation between PHR and aneurysm diameter in patients with AAA. **(A)** Correlation between PHR and aneurysm diameter in patients with AAA; **(B)** The correlation between HDL-C and platelet count in patients with AAA; **(C)** The correlation between HDL-C and aneurysm diameter in AAA patients; **(D)** The correlation between platelet count and aneurysm diameter in AAA patients. PHR, platelet to high-density lipoprotein cholesterol ratio; AAA, abdominal aortic aneurysm; HDL-C, high density lipoprotein cholesterol.

To further evaluate whether the PHR is associated with the disease progression in patients with AAA, we further examined the correlation between PHR and the diameter of AAA. The results showed that there was a significant positive correlation between the PHR level and the aneurysm diameter in AAA patients (*r* = 0.413, *P* < 0.001) (Figure 2A). This suggests that PHR has potential value in assessing the progression of AAA. Additionally, we conducted a detailed analysis of the correlation between HDL-C and platelet count in patients with AAA. Our results showed no significant correlation between these two variables (*r* = 0.076, *P* = 0.217) (Figure 2B), suggesting that HDL-C and platelets do not have a substitutable relationship. Further correlation analysis between HDL-C and aneurysm diameter in AAA patients revealed a significant negative correlation (*r* = −0.194, *P* = 0.015) (Figure 2C), which implies that lower HDL-C levels may be associated with aneurysm expansion. In contrast, a significant positive correlation was observed between platelet count and aneurysm diameter in AAA patients (*r* = 0.334, *P* < 0.001) (Figure 2D), indicating that higher platelet counts may be associated with aneurysm expansion.

### 3.3 The association between PHR and thrombosis in AAA patients

**Figure 3:**
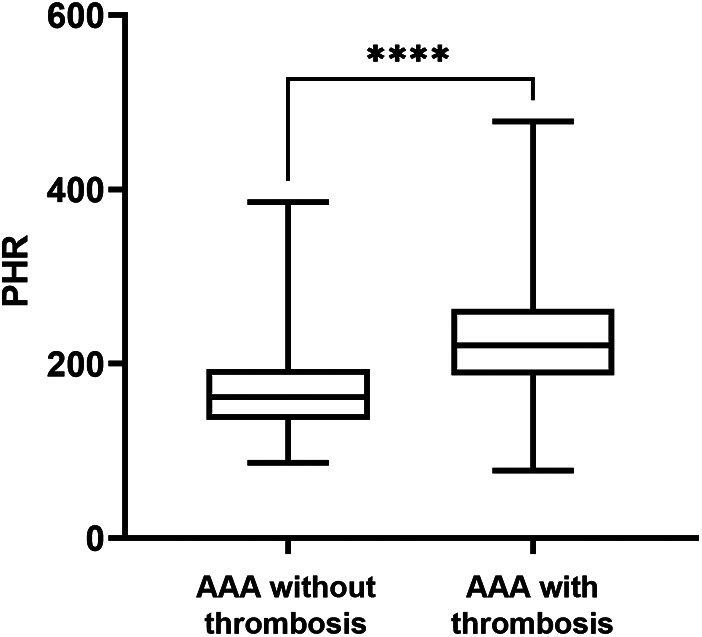
PHR levels in AAA patients divided by the thrombosis. Data were presented as median (min -max). AAA, abdominal aortic aneurysm; PHR, platelet to high-density lipoprotein cholesterol ratio; ****P < 0.0001.

Among the 156 patients with AAA, a total of 80 patients had concomitant thrombosis. Subsequently, we further analyzed the differences in PHR between AAA patients with thrombosis and those without thrombosis. The results indicated that the PHR in AAA patients with thrombosis was significantly higher than that in AAA patients without thrombosis [220.61(186.49, 262.27) vs. 161.80(135.54, 193.41); *P* < 0.001] (Figure 3). Moreover, there were more smokers among AAA patients with thrombosis, and they had higher platelet counts, higher TG and LDL-C levels, and lower HDL-C levels (Table 2).

**Table 2:** Clinical characteristics of AAA patients grouped by thrombosis.

Variables	AAA without thrombosis	AAA with thrombosis	*P* value
General characteristics			
Age (years)	60.55 ± 9.66	60.24 ± 8.49	0.829
Male, *n* (%)	66 (86.8)	69 (86.2)	0.914
BMI (kg/m^2^)	23.27 ± 2.45	24.05 ± 2.55	0.083
Heart rate (beats per minute)	79.10 ± 13.88	80.94 ± 12.87	0.394
Comorbidities			
CHD, *n* (%)	10 (13.2)	14 (17.5)	0.452
Smoking, *n* (%)	22 (28.9)	37 (46.2)	0.026
Hypertension, *n* (%)	45 (59.2)	51 (63.8)	0.560
Diabetes, *n* (%)	9 (11.8)	9 (11.3)	0.908
Laboratory values			
PHR	161.80 (135.54,193.41)	220.61 (186.49, 262.27)	<0.001
Platelet count (×10^9^/L)	161.50 (142.25, 199.00)	208.00 (181.00, 254.00)	<0.001
HDL-C (mmol/L)	1.08 ± 0.27	0.97 ± 0.20	0.006
TG (mmol/L)	1.09 (0.81, 1.46)	1.63 (1.13, 2.77)	<0.001
TC (mmol/L)	4.52 ± 1.33	4.42 ± 1.22	0.608
LDL-C (mmol/L)	2.53 ± 1.03	2.96 ± 1.08	0.012
WBC count (×10^9^/L)	6.13 (5.19, 7.55)	6.71 (5.51, 8.20)	0.142
Serum creatinine (μmol/L)	80.90 (67.90, 95.80)	81.70 (67.20, 101.60)	0.872
Uric acid (μmol/L)	341.60 (297.85, 451.55)	344.70 (299.80, 412.90)	0.741
BUN (mmol/L)	6.35 (5.25, 7.85)	6.25 (4.77, 7.43)	0.193

AAA, abdominal aortic aneurysm; BMI, body mass index; CHD, coronary heart disease; PHR, platelet to high-density lipoprotein cholesterol ratio; HDL-C, high density lipoprotein cholesterol; TG, triglyceride; TC, total cholesterol; LDL-C, low density lipoprotein cholesterol; WBC, white blood cell; BUN, blood urea nitrogen.

### 3.4 Evaluation of the diagnostic value of PHR for AAA

**Figure 4:**
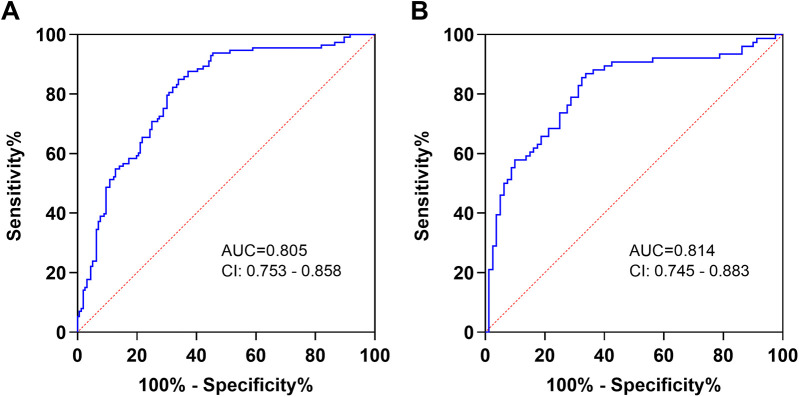
Diagnostic value of PHR in AAA patients. **(A)** The ROC curves of PHR to AAA patients and healthy controls. **(B)** The ROC curves of PHR to thrombosis in AAA patients. AUC, area under the curve; CI, confidence interval.

To further evaluate the diagnostic performance of PHR in patients with AAA, we conducted a ROC curve analysis. The results showed that PHR had a good predictive value for the diagnosis of AAA. The area under the curve (AUC) was 0.805, and the optimal cut-off value was 170.7 (sensitivity: 84.96%, specificity: 66.03%) (Figure 4A). In addition, for the diagnosis of AAA complicated with thrombosis, the AUC of PHR was 0.814, and the optimal cut-off value was 204.6 (sensitivity: 86.84%, specificity: 66.25%) (Figure 4B). To assess whether the PHR offers superior diagnostic performance compared to platelet count and HDL-C individually, we conducted ROC curve analyses for both platelet count and HDL-C. Our findings revealed that HDL-C also holds considerable diagnostic value for AAA. However, when considering the overall diagnostic capabilities, PHR not only exhibited robust diagnostic efficacy for AAA but also significantly outperformed both platelet count and HDL-C in diagnosing AAA complicated with thrombosis (Additional File 1: Supplementary Figure S2). Furthermore, to determine if PHR surpasses traditional factors in diagnosing AAA, we compared the diagnostic performance of PHR alone, hypertension alone, and the combination of PHR and hypertension using ROC curves. The results indicated that PHR alone exhibits superior diagnostic performance compared to hypertension. Moreover, integrating PHR with hypertension further enhances diagnostic accuracy (Additional File 1: Supplementary Figure S3).

### 3.5 Univariate and multivariate logistic regression analyses

To investigate whether PHR could serve as a predictor for AAA, univariate and multivariate logistic regression analyses were performed, and the results are shown in Table 3. Univariate logistic regression analysis revealed that age, heart rate, hypertension, PHR, HDL-C, TG, LDL-C, WBC, serum creatinine, and BUN were significantly associated with AAA. Subsequently, multivariate regression analysis indicated that PHR (OR: 1.022; 95% CI: 1.012–1.031; *P* < 0.001) remained an independent predictor of AAA after adjusting for confounding factors. Additionally, hypertension, serum creatinine, and BUN were also associated with the presence of AAA.

**Table 3:** Univariate and multivariate logistic regression analysis showing the independent predictors for AAA.

Variables	Univariate analysis OR (95% CI)	*P* value	Multivariate analysis OR (95% CI)	*P* value
Age	1.027 (1.000–1.055)	0.050	1.005 (0.961–1.052)	0.816
Male	0.643 (0.334–1.238)	0.187		
BMI	1.029 (0.927–1.142)	0.592		
Heart rate	1.032 (1.011–1.053)	0.003	1.015 (0.976–1.054)	0.459
CHD	1.686 (0.789–3.601)	0.177		
Smoking	1.413 (0.844–2.368)	0.189		
Hypertension	7.916 (4.392–14.268)	<0.001	4.024 (1.732–9.354)	0.001
Diabetes	0.922 (0.438–1.942)	0.832		
PHR	1.024 (1.017–1.030)	<0.001	1.022 (1.012–1.031)	<0.001
Platelet count	1.002 (0.997–1.006)	0.417		
HDL-C	0.002 (0.000–0.008)	<0.001	/	
TG	1.490 (1.122–1.980)	0.006	1.104 (0.886–1.376)	0.378
TC	1.047 (0.834–1.316)	0.691		
LDL-C	1.529 (1.124–2.082)	0.007	1.268 (0.769–2.090)	0.352
WBC count	1.319 (1.142–1.523)	<0.001	1.016 (0.769–1.343)	0.910
Serum creatinine	1.032 (1.014–1.051)	0.001	1.036 (1.010–1.063)	0.007
Uric acid	1.000 (0.999–1.001)	0.490		
BUN	1.410 (1.148–1.733)	0.001	1.400 (1.051–1.866)	0.021

AAA, abdominal aortic aneurysm; BMI, body mass index; CHD, coronary heart disease; PHR, platelet to high-density lipoprotein cholesterol ratio; HDL-C, high density lipoprotein cholesterol; TG, triglyceride; TC, total cholesterol; LDL-C, low density lipoprotein cholesterol; WBC, white blood cell; BUN, blood urea nitrogen; OR, odds ratio; CI, confidence interval.

We further explored the predictive value of PHR for thrombosis in AAA patients, and the results are shown in Table 4. Univariate logistic regression analysis revealed that smoking, PHR, platelet count, HDL-C, TG, and LDL-C were closely associated with thrombosis. After adjusting for univariate predictors with *P* < 0.10, PHR (OR: 1.016; 95% CI: 1.007–1.024; *P* < 0.001) remained an independent factor for predicting thrombosis in AAA patients.

**Table 4:** Univariate and multivariate logistic regression analysis showing the independent predictors for thrombosis in AAA patients.

Variables	Univariate analysis OR (95% CI)	*P* value	Multivariate analysis OR (95% CI)	*P* value
Age	0.996 (0.962–1.031)	0.827		
Male	1.052 (0.419–2.641)	0.914		
BMI	1.137 (0.982–1.316)	0.086	1.114 (0.936–1.325)	0.224
Heart rate	1.010 (0.987–1.035)	0.392		
CHD	1.400 (0.581–3.376)	0.454		
Smoking	2.112 (1.089–4.097)	0.027	2.008 (0.832–4.850)	0.121
Hypertension	1.211 (0.635–2.311)	0.560		
Diabetes	0.944 (0.353–2.520)	0.908		
PHR	1.020 (1.012–1.027)	<0.001	1.016 (1.007–1.024)	<0.001
Platelet count	1.017 (1.010–1.025)	<0.001	/	
HDL-C	0.143 (0.034–0.602)	0.008	/	
TG	1.828 (1.254–2.665)	0.002	1.611 (1.068–2.429)	0.023
TC	0.936 (0.730–1.202)	0.606		
LDL-C	1.504 (1.081–2.093)	0.016	1.275 (0.857–1.898)	0.230
WBC count	1.092 (0.961–1.240)	0.176		
Serum creatinine	1.002 (0.990–1.014)	0.765		
Uric acid	1.000 (0.997–1.002)	0.736		
BUN	0.890 (0.773–1.024)	0.103		

AAA, abdominal aortic aneurysm; BMI, body mass index; CHD, coronary heart disease; PHR, platelet to high-density lipoprotein cholesterol ratio; HDL-C, high density lipoprotein cholesterol; TG, triglyceride; TC, total cholesterol; LDL-C, low density lipoprotein cholesterol; WBC, white blood cell; BUN, blood urea nitrogen; OR, odds ratio; CI, confidence interval.

## 4 Discussions

In this study, we have demonstrated that the PHR is significantly elevated in patients with AAA, particularly in those with thrombosis, compared to controls. Importantly, we have identified a positive correlation between PHR and aneurysm diameter in AAA patients. Furthermore, our analysis indicates that PHR serves as an independent predictor of both AAA and AAA complicated with thrombosis. Specifically, at a cutoff value of 170.7, PHR achieves a sensitivity of 84.96% and a specificity of 66.03% for identifying AAA patients. At a higher cutoff value of 204.6, the sensitivity increases to 86.84%, with a specificity of 66.25% for detecting thrombosis in AAA patients. To our knowledge, this study represents the first to provide compelling evidence supporting the potential use of PHR as a novel diagnostic marker for AAA.

Studies have consistently shown that HDL-C levels are inversely related to the risk of AAA (13). For instance, a Danish registry study revealed that the average HDL-C concentration in AAA patients was significantly lower than that in patients with aorto-iliac occlusive diseases (25). Furthermore, there is a clear inverse correlation between HDL-C levels and AAA diameter, and therapies targeting HDL-C have been shown to effectively contribute to the prevention of AAA formation (26). HDL-C is primarily synthesized in the liver and is responsible for transporting cholesterol from extra-hepatic tissues to the liver for excretion (27). In AAA patients, however, the ability of HDL-C to promote cholesterol efflux from macrophages in the aortic wall is impaired (28). As a result, macrophages in the aortic wall of AAA patients become engorged with cholesterol, and the normal function of HDL-C in removing this cholesterol is disrupted, which may contribute to the development of AAA (28). Moreover, reduced levels of HDL-C can lead to a weakening of its anti-inflammatory, anti-oxidative, and anti-atherosclerotic effects, thereby promoting the occurrence and progression of AAA (27, 29).

In addition to abnormal lipid metabolism, hypertension—a traditional risk factor for AAA—also plays a significant role in the diagnosis of AAA. Our findings highlight that combining the PHR with hypertension significantly enhances diagnostic performance. This improvement can be explained by the following mechanisms: hypertension primarily damages the vascular wall structure through sustained blood pressure elevation, yet it fails to specifically identify other pathophysiological aspects of AAA. Conversely, PHR integrates platelet-driven inflammatory responses and HDL-C mediated anti-atherosclerotic effects, can precisely encapsulate the central pathological shifts in AAA's evolution and progression. This unique mechanistic profile empowers PHR to function not only as an independent AAA predictor but also to refine diagnostic precision when coupled with traditional risk factors like hypertension, thereby furnishing a more holistic foundation for the precise identification of AAA.

Previous studies have demonstrated that platelet count is closely associated with poor postoperative prognosis in patients with ruptured AAA (30). Specifically, patients with decreased platelet counts are at a significantly increased risk of bleeding and multiple organ failure, while those with elevated platelet levels are more prone to developing deep venous thrombosis and pulmonary embolism (30). In our current study, although no significant difference in platelet count was observed between AAA patients and healthy controls, the PHR was significantly elevated in AAA patients, irrespective of their platelet count levels. Moreover, a positive correlation was established between platelet count and aneurysm diameter, underscoring the close relationship between platelet count and the severity of AAA. This finding contrasts with previous studies that reported a significant decrease in platelet count among patients with ruptured AAA (30). The discrepancy can be attributed to the differing study populations. Our study predominantly included patients without acute rupture, resulting in relatively mild platelet consumption. Conversely, the prior study centered on patients undergoing surgical repair for ruptured AAA, a context where platelet activation and consumption are more pronounced, thereby increasing the likelihood of a reduced platelet count. This distinction in study populations accounts for the divergent findings regarding platelet count.

In addition, our study also found that the PHR level was higher in AAA patients with thrombosis. Moreover, the ROC curve indicated that PHR had a relatively high diagnostic value for thrombosis in AAA patients. Multivariate regression analysis showed that PHR was an independent predictor of thrombosis in AAA patients. Thrombosis is very common in AAA patients (31). The role of thrombosis in the progression of AAA is complex, but overall, its harmful effects outweigh its protective effects (32). Studies have shown that intraluminal thrombosis is closely associated with the rapid growth of AAA (33). Therefore, early screening for thrombosis in AAA patients is crucial for patient risk stratification and treatment.

Platelets and HDL-C are closely related to thrombosis in AAA. Platelets are of great significance in the process of formation and the growth of the thrombosis in AAA. Platelets play a crucial role in the formation and growth of thrombosis in AAA. Thrombosis occurs when platelets adhere to the complex formed by collagen and von Willebrand factor (vWF) (32). Subsequently, the binding of fibrinogen to activated integrin αIIbβ3 on platelets leads to platelet aggregation (32). Moreover, platelets within the thrombus, which have secretory functions, release soluble CD40 ligand (sCD40l), soluble P-selectin, and other substances into the bloodstream (34). These released substances can enhance the activation and accumulation of inflammatory cells (35), thereby accelerating the development of both thrombus and AAA. In addition to platelets, HDL-C also plays an important regulatory role in thrombosis. Studies suggest that HDL-C exerts an antithrombotic effect by preventing the self-association of vWF and the subsequent adhesion of platelets (36). Our study demonstrates that the PHR, a composite index composed of platelets and HDL-C, has a relatively high diagnostic value for thrombosis in AAA patients. However, due to the small sample size in this study, further verification by large-sample clinical studies is still needed.

The positive correlation between PHR and AAA diameter observed in our study is consistent with prior research linking low HDL-C levels to the formation and progression of AAA (13, 37). HDL-C deficiency promotes macrophage infiltration and foam cell formation in the aortic wall, while platelet hyperactivity accelerates intraluminal thrombosis formation (28, 32, 33). The PHR metric integrates these dual pathways: low HDL-C fosters a microenvironment conducive to inflammation, while elevated platelet counts exacerbate thrombus-mediated injury. This synergy likely amplifies extracellular matrix degradation and aneurysmal dilation. Clinically, the correlation between PHR and aneurysm diameter suggests that systemic metabolic-inflammatory derangements, rather than localized pathology alone, drive AAA growth. The predictive utility of the PHR may outperform traditional cardiovascular risk factors due to its composite reflection of the pro-thrombotic and anti-inflammatory imbalance (38, 39).

Although the diagnostical performance of PHR in AAA is encouraging, there are several limitations that need to be mentioned. Firstly, the retrospective, single-center design with a small sample size limits causal inference and accuracy, necessitating validation in larger, prospective cohorts. Secondly, the present study primarily focuses on the diagnostic value of the PHR in patients with AAA. It did not encompass long-term follow-up of AAA patients, nor did it evaluate the impact of PHR on the prognosis of AAA patients. Future research endeavors will involve extended follow-up of AAA patients to explore the correlation between PHR and acute AAA rupture, as well as to monitor the dynamic changes in PHR levels following treatment. Thirdly, non-invasive imaging at diagnosis can directly visualizes the presence of intraluminal thrombus along with aneurysm diameter in AAA patients. However, frequent follow-up imaging is often impractical, making it challenging to dynamically monitor the formation of intraluminal thrombus in AAA patients and to assess the dynamic process of thrombus development. In contrast, the PHR can be readily calculated from routine blood test results, offering the advantages of ease of use and low cost. This biomarker not only facilitates follow-up but also enables dynamic monitoring and assessment of thrombus formation in AAA patients, thus serving as a practical alternative or supplementary tool to imaging examinations.

## 5 Conclusion

Our findings reveal that the PHR possesses substantial diagnostic value for patients with AAA. Notably, PHR maintains robust diagnostic performance even in AAA patients with thrombus formation. These results suggest that PHR has the potential to serve as a practical and scalable clinical tool for the diagnosis of AAA.

## Data Availability

The original contributions presented in the study are included in the article/Supplementary Material, further inquiries can be directed to the corresponding author/s.
