# Supplementary material for: Diagnostic value of platelet to high-density lipoprotein cholesterol ratio in abdominal aortic aneurysms
Source: Front Cardiovasc Med. 2025 Oct 30;12:1687265. doi: 10.3389/fcvm.2025.1687265 (PMC12611948; doi:10.3389/fcvm.2025.1687265)
Supplement: Supplementary file 1 [file Datasheet1.pdf]

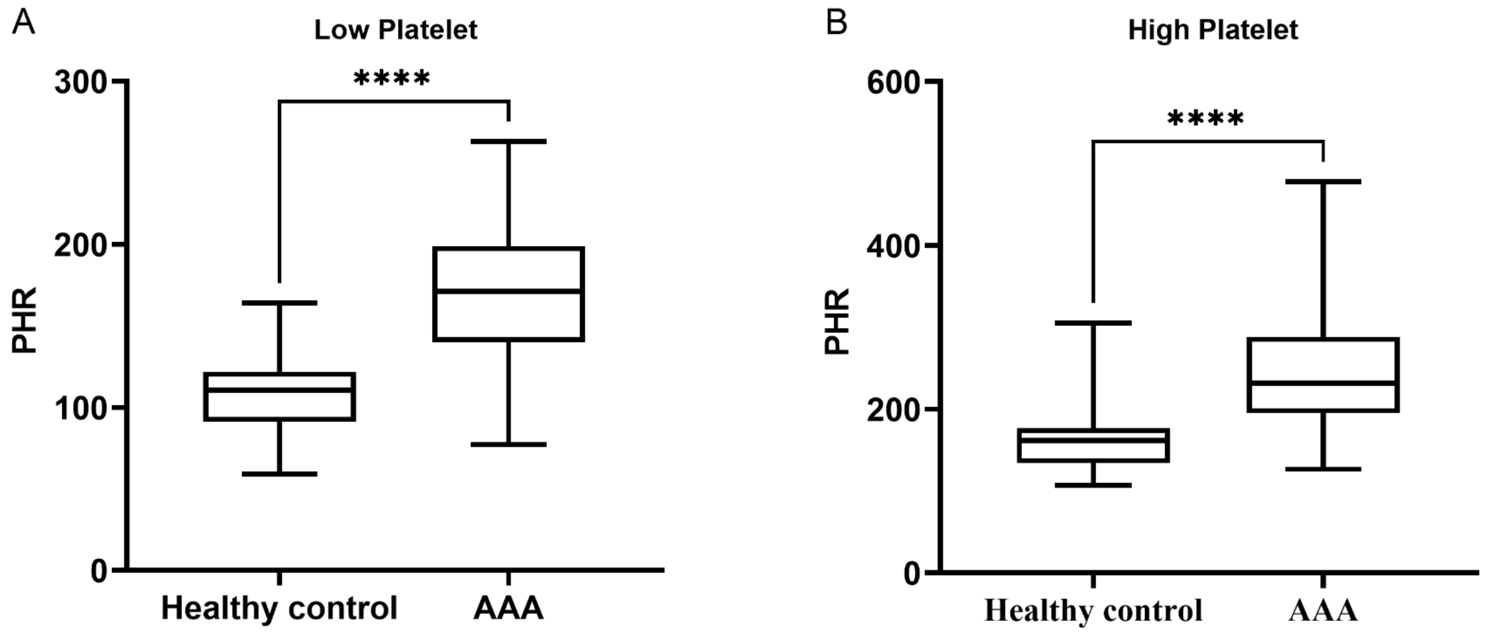

Figure S1. PHR levels in AAA and healthy patients stratified by platelet count.

Patients with AAA and healthy controls were divided into a high platelet group (above the average platelet count) and a low platelet group (below the average platelet count) based on the average platelet count of each group. Data were presented as median (min-max). AAA: abdominal aortic aneurysm; PHR: platelet to high-density lipoprotein cholesterol ratio; ****p < 0.0001.

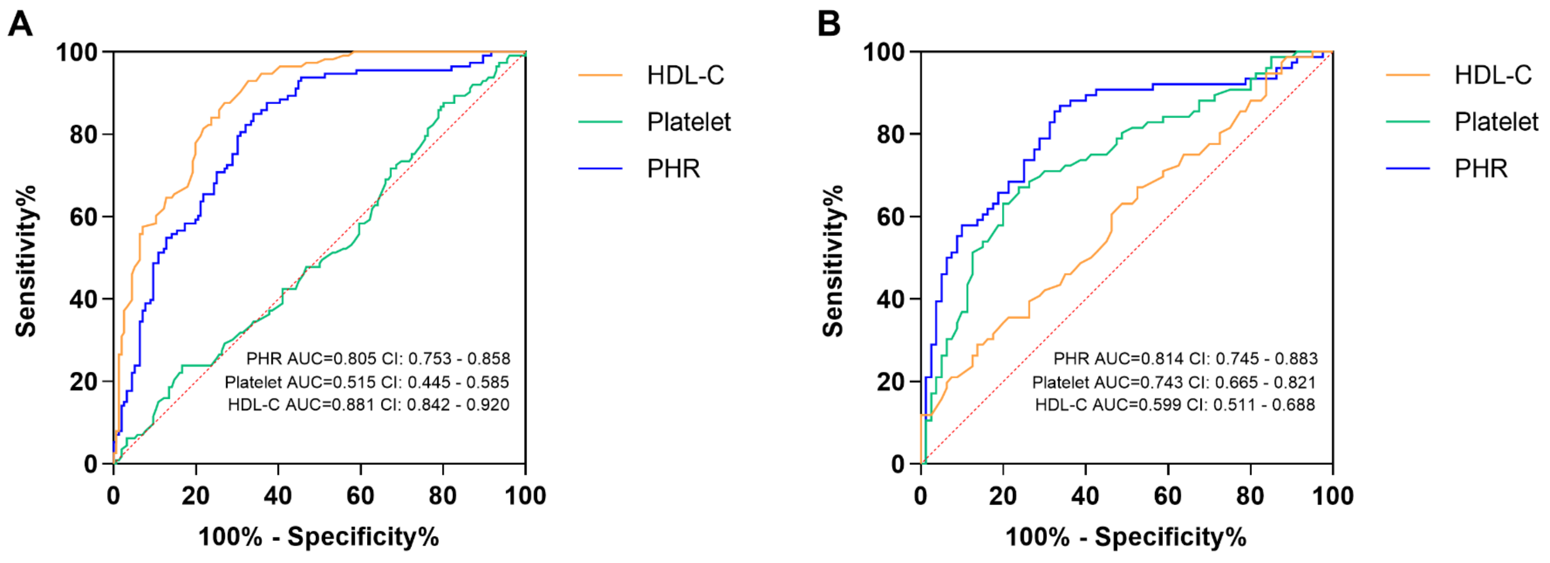

Figure S2. diagnostic value of HDL-C, Platelet, and PHR in AAA patients.

(A) The ROC curves of HDL-C, Platelet, and PHR to AAA patients and healthy controls. (B) The ROC curves of HDL-C, Platelet, and PHR to thrombosis in AAA patients. HDL-C: high-density lipoprotein cholesterol; PHR: platelet to high-density lipoprotein cholesterol ratio; AUC: area under the curve; CI: confidence interval.

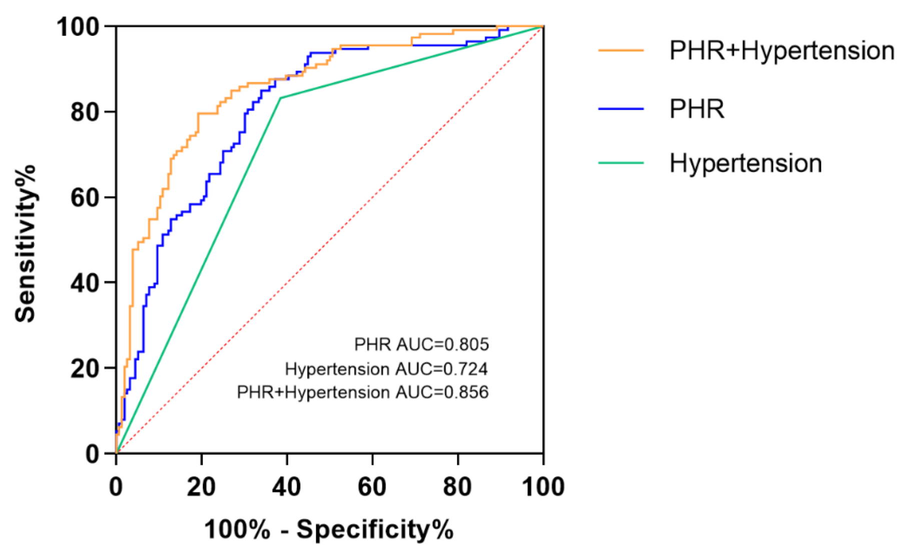

Figure S3. diagnostic value of PHR, hypertension, and PHR combined with hypertension in AAA patients.

PHR: platelet to high-density lipoprotein cholesterol ratio; AUC: area under the curve; CI: confidence interval.
